# Reassessing pregnancy intention and its relation to maternal, perinatal and neonatal outcomes in a low-income setting: A cohort study

**DOI:** 10.1371/journal.pone.0205487

**Published:** 2018-10-18

**Authors:** Jennifer Anne Hall, Geraldine Barrett, Andrew Copas, Tambosi Phiri, Address Malata, Judith Stephenson

**Affiliations:** 1 Research Department of Reproductive Health, UCL Institute for Women’s Health, London, United Kingdom; 2 Department of Infection & Population Health, UCL Institute of Epidemiology and Health Care, London, United Kingdom; 3 MaiMwana Project, Mchinji, Malawi; 4 Malawi University of Science and Technology, Zomba, Malawi; Public Health Foundation of India, INDIA

## Abstract

**Background:**

It is unclear whether unintended pregnancies are associated with adverse outcomes. Data are predominantly from high-income countries and have methodological limitations, calling the findings into question. This research was designed to overcome these limitations and assess the relationships between pregnancy intention and miscarriage, stillbirth, low birthweight, neonatal death and postnatal depression in a low-income country.

**Methods:**

The pregnancy intention of 4,244 pregnant women in Mchinji District, Malawi, was measured using the validated Chichewa version of the London Measure of Unplanned Pregnancy (LMUP). Women were re-interviewed postnatally to assess pregnancy outcome. Postnatal depression was assessed using the WHO’s Self-Reporting Questionnaire. Multivariable regressions were conducted, with the choice of confounders informed by a pre-existing conceptual epidemiological hierarchy.

**Results:**

Planned pregnancies are associated with a reduced risk of any (adjusted RR 0.90 [95%CI 0.86, 0.95]) or high symptoms of depression (adjusted RR 0.76 [95%CI 0.63, 0.91]) compared to unplanned pregnancies in rural Malawi. There was no relationship between pregnancy intention and the composite measure of miscarriage, stillbirth, low birthweight and neonatal death. There was some evidence that greater pregnancy intention was associated with reduced adjusted risk of stillbirth (0·93 [95%CI 0·87, 1·00]).

**Conclusion:**

Our study is the first to use a psychometrically valid measure of pregnancy intention, and to do so antenatally. As pregnancy intention increases, the risk of postnatal depression and, possibly, stillbirth decreases. This suggests a new, clinical use for the LMUP; identifying women antenatally who are at risk of these adverse pregnancy outcomes.

## Introduction

Unintended pregnancies may pose an increased risk to both mother and baby but the evidence on this is inconclusive [1], largely due to the methodological problems faced in the investigation of relationships between pregnancy intention and maternal and child outcomes. Two key challenges are accurate assessment of pregnancy intention and adequate adjustment for confounding factors.

Most estimates of the levels of unintended pregnancy in low-income countries are derived from surveys such as the Demographic and Health Survey (DHS). This asks ‘At the time you became pregnant did you want to become pregnant then, did you want to wait until later, or did you want to have no (more) children at all?’. These questions were devised before psychometric, or latent variable measurement methods, were widely adopted in health research and therefore classify pregnancies as intended or unintended on the basis of a single question, with the inherent measurement error that will involve. In recent decades there has been discussion of the limitations of the DHS style methodology and of the need for a more sophisticated way of measuring pregnancy intention [2–9]. In response, the London Measure of Unplanned Pregnancy (LMUP), a psychometrically validated measure of the degree of intention of a current or recent pregnancy, has been developed [3]. The LMUP was designed following extensive qualitative work with pregnant women to map the construct of pregnancy intention. The ensuing conceptual framework guided the development of items which were then piloted, field-tested and psychometrically evaluated to finalise the content of the LMUP. This groundwork and measure development methodology mean that the LMUP should be a more accurate and valid way of measuring pregnancy intentions. It is being used internationally [10–15] but, despite being the only validated measure, has not yet superseded the DHS-type questions that most studies continue to use.

In DHS and similar surveys, questions about pregnancy intention are asked of women up to five years after the birth of a baby. This potentially introduces recall bias and overestimates intention because reported intention tends to increase after delivery [16] and because miscarriages and induced abortions are omitted. Moreover, retrospective assessment of pregnancy intention means that reported intention can be influenced by the outcome of pregnancy [17], making distinction between cause and effect impossible.

Previous studies in this area have been inconsistent regarding which confounders are controlled for, if any. For example, in six studies examining unintended pregnancy and low birthweight (LBW) no two studies controlled for the same combination of ‘confounders’, which ranged from socio-demographics and obstetric history to uptake of antenatal care [18–23]. The findings of these studies were inconsistent. Consequently considerable uncertainty remains as to whether pregnancy intention is independently associated with maternal, perinatal or neonatal outcomes.

The aim of this study is to fill the gap in our knowledge of relationships between pregnancy intention and maternal, perinatal and neonatal health outcomes in a low-income country setting. It overcomes previous methodological limitations by using the LMUP (a validated measure) to assess intention during pregnancy and by following women up after the end of the neonatal period to assess outcomes (to reduce recall bias and prevent pregnancy outcome from influencing the reported intention). We collected data on a wide range of potential confounders and used previous evidence to develop a conceptual epidemiology hierarchy of the determinants of pregnancy intention to inform our choice of variables in our multiple regression analyses. We hypothesised that unplanned pregnancies would be associated with worse outcomes for mother and baby.

## Methods

### Study setting and design

The study was conducted in Malawi, a land-locked country in sub-Saharan Africa. Malawi is one of the least developed countries in the world, ranking 170^th^ out of 188 countries on the most recent Human Development Index [24]. At the time of this study (2012–14) the maternal mortality ratio and under five mortality rates were high (675 maternal deaths per 100 000 live births and 112 child deaths per 1000 live births respectively) [25]. The total fertility rate was 5.7 children per woman and 26% of married women had an unmet need for family planning [25]. Recent estimates suggest that 53% of pregnancies are unplanned, and that 30% of these end in abortion (around 141,000 abortions in 2015 [26]), though this is illegal. Abortions cause between 6–18% of maternal deaths in Malawi [27], meaning unplanned pregnancy is an important contributor to maternal death.

The high total fertility rate, large proportion of unintended pregnancies and high levels of mortality make Malawi an excellent setting for this research. Mchinji District is a rural district in Malawi with a population of over 530 000, 23% of whom are women of child bearing age (121 950). Around 90% of the population are subsistence farmers. In Mchinji the total fertility rate was 6.3 children per woman, yet the total wanted fertility rate was 4.6 children per woman, and there was a high unmet need for family planning (29.3% in married women) [25] suggesting that there was a high proportion of unplanned pregnancies locally.

Family planning services are provided free of charge in Malawi through government health facilities, and are available for purchase through private clinics such as ‘Banja la Mtsogolo’, a Marie Stopes International Partner. There are 14 health facilities in Mchinji District; one District Hospital, one Mission Hospital, three community hospitals and nine health centres. On average women live almost 6km from the nearest health facility; a distance they would likely have to cover on foot, presenting time, travel and opportunity costs associated with accessing family planning.

Previous research divided Mchinji District into 49 geographical areas [28]; from this sampling frame a random sample of 25 areas were selected. Using the pre-existing district-wide surveillance system, all pregnant women aged 15 and over in these areas were identified between March and December 2013. A trained data collector visited the pregnant women at home, gained written informed consent and conducted a 20-minute interview. Women were eligible to participate at any point during their pregnancy. Pregnancy intention was measured using the validated Chichewa version of the LMUP [29]. There are six questions in the LMUP, covering contraceptive use, timing of pregnancy, intention, desire for a baby, partner discussions and pre-pregnancy preparations. Each question is scored zero, one or two, thereby producing a score on an ordinal scale of zero to 12. Each increase in score reflects an increase in the level pregnancy intention. In the absence of a validated tool for assessing previous depression, we worked with experts in the field to devise four questions which were used to categorise women as to the extent of possible previous depression. Women who experienced both low mood and anhedonia for more than two weeks in the year before they became pregnant were considered most likely to have experienced previous depression (labelled as ‘both ≥2 weeks’ in the tables). Those who experienced only one of these (one ≥ 2 weeks), or who experienced them for a period of less than two weeks (one/both < 2 weeks), were considered less likely to have experienced previous depression and women who reported neither of these were the least likely to have experienced previous depression. Intimate partner violence was assessed using the Abuse Assessment Screen [30]. This asks about experience of emotional or physical abuse ever, in the last year or while pregnant, as well as experience of sexual abuse.

Follow-up was conducted between May 2013 and July 2014. Women were re-visited at least 28 days after the end of the pregnancy to collect data on the outcome of the pregnancy. The outcome of interest for the mother was postnatal depression which was assessed using the validated Chichewa version of the World Health Organization’s 20-question screening tool, the Self-Reporting Questionnaire (SRQ) [31]. This asks about the presence or absence of some symptoms of depression and anxiety in the preceding four weeks. For the child we used a composite adverse pregnancy outcome measure of miscarriage (pregnancy lost before 28 weeks’ gestation), stillbirth (baby born with no signs of life at or after 28 weeks' gestation), LBW (baby born weighing less than 2,500g regardless of gestation) or neonatal death (baby born alive but who dies within the first 28 days of life). No distinction was made between ante- and intrapartum stillbirth as we had no reliable way to collect this information. These outcomes were chosen because of their importance for maternal, perinatal and neonatal health, their higher prevalence in low-income settings, the theoretical basis for a role of pregnancy intention in influencing these outcomes and the feasibility of collecting reliable data on them.

### Sample size

We estimated that 41% of pregnancies would be unplanned [32], there would be a prevalence of 15% for both postnatal depression and the composite adverse pregnancy outcome variable [25, 33–35] and a relative risk of 1.25 (prevalence of adverse outcomes 13.6% in planned and 17.0% in unplanned pregnancies) [36]. For 80% power at the 0.05 significance level the power calculation indicated that 3,737 pregnancy outcomes were needed.

### Statistical analysis

Multivariable relative risk regression was used for the binary adverse pregnancy outcome variable, specifically a generalised linear model was fitted with a log link function. Analysis of each individual outcome was also conducted to look for relationships obscured by the composite variable, due to the fact that low birth weight was significantly more common than the other outcomes, although the power of these individual outcome analyses is limited.

The distribution of the antenatal LMUP score was non-Normal, as is seen elsewhere, [3, 11, 12, 37–39] and the SRQ score was positively skewed. The relationship between the antenatal LMUP score and the postnatal SRQ score was non-linear. We investigated transformations, including box-cox for the SRQ score and fractional polynomials for the antenatal LMUP score, but the distribution of the resulting residuals still violated the assumptions of linear regression so that this method was not used. Next we tried grouping the SRQ score and conducted ordinal logistic regressions but were not able to fit a model that did not violate model assumptions. We therefore created two binary variables for the SRQ score based on the cut-points used by Hanlon et al [40] and developed separate multivariable relative risk regression models at each cut-point using Poisson regression. The cut-points were between 0/1 (no symptoms of depression / any symptoms of depression) and between 5/6 (no or any symptoms of depression / high symptoms of depression). We also collapsed the antenatal LMUP score into the recognised groupings of 0–3 (unplanned), 4–9 (ambivalent) and 10–12 (planned) [3].

The variables considered for inclusion in the models (Table 1) were based on our previous findings of the determinants of pregnancy intention in this setting, which used an a priori conceptual epidemiological hierarchy [41]. Women in the study were interviewed between two and nine months’ gestation, but the timing of assessment was not found to affect reported intention in the final model of the determinants of pregnancy intention [41]. Variables found to be associated with pregnancy intention on that analysis were considered for selection in these multivariable models if their univariate relationship with the primary outcome was significant at p<0·1. Variables were selected for inclusion in the final model following a manual backwards-stepwise approach using a threshold of p<0.05. Clustering of participants by geographical area was acknowledged by presenting robust standard errors. All analyses were conducted in Stata 13 (StataCorp. 2013. Stata Statistical Software: Release 13. College Station, TX: StataCorp LP.)

**Table 1:** Determinants of pregnancy intention considered for inclusion in the multivariable models.

Variables considered for inclusion in multivariable models	
Socio-economic status	Primiparity
Woman's education	Partner's age
Number of live children	Woman's age
Marital status	Geographical area
Intimate partner violence	Time since last birth
Previous episodes of depression	

### Missing data

There were few missing data for antenatal LMUP score (19 women, <0·5%) or postnatal SRQ score (39 women, 0·9%); these were imputed using mean imputation. Given the small numbers this was not expected to introduce bias but the models were rechecked on the non-imputed data as a sensitivity analysis. There was 22% missing data for birthweight. Babies that were stillborn or who were born at home or in transit were significantly less likely to have been weighed at birth, suggesting the data were ‘missing at random’ (ie the missingness can be explained by other variables). A multiple imputation model using an iterative Markov chain Monte Carlo method, including anthropometric measurements from the child and the mother (see S1 Table), was used to impute missing birthweight data. Twenty imputed datasets were created. There were no missing data for miscarriage, stillbirth or neonatal death, except from two maternal deaths where the baby’s outcome could not be ascertained.

### Ethical approval

The UCL Research Ethics Committee and the College of Medicine Research Ethics Committee at the University of Malawi granted ethical approval for this research, reference numbers 3974/001 and P.03/12/1273 respectively. All participants gave written informed consent to take part in this research, by thumbprint if necessary, after they had read the information sheet and/or had the study explained to them. The participants retained the information sheet and one copy of the signed consent form; a second copy of the signed consent form was stored in a lockable cabinet in the main study office. Both ethics committees approved this consent procedure. Local approval to conduct the research in Mchinji District was given by the District Health Officer and the District Executive Committee.

## Results

### Recruitment and loss to follow-up

**Fig 1:**
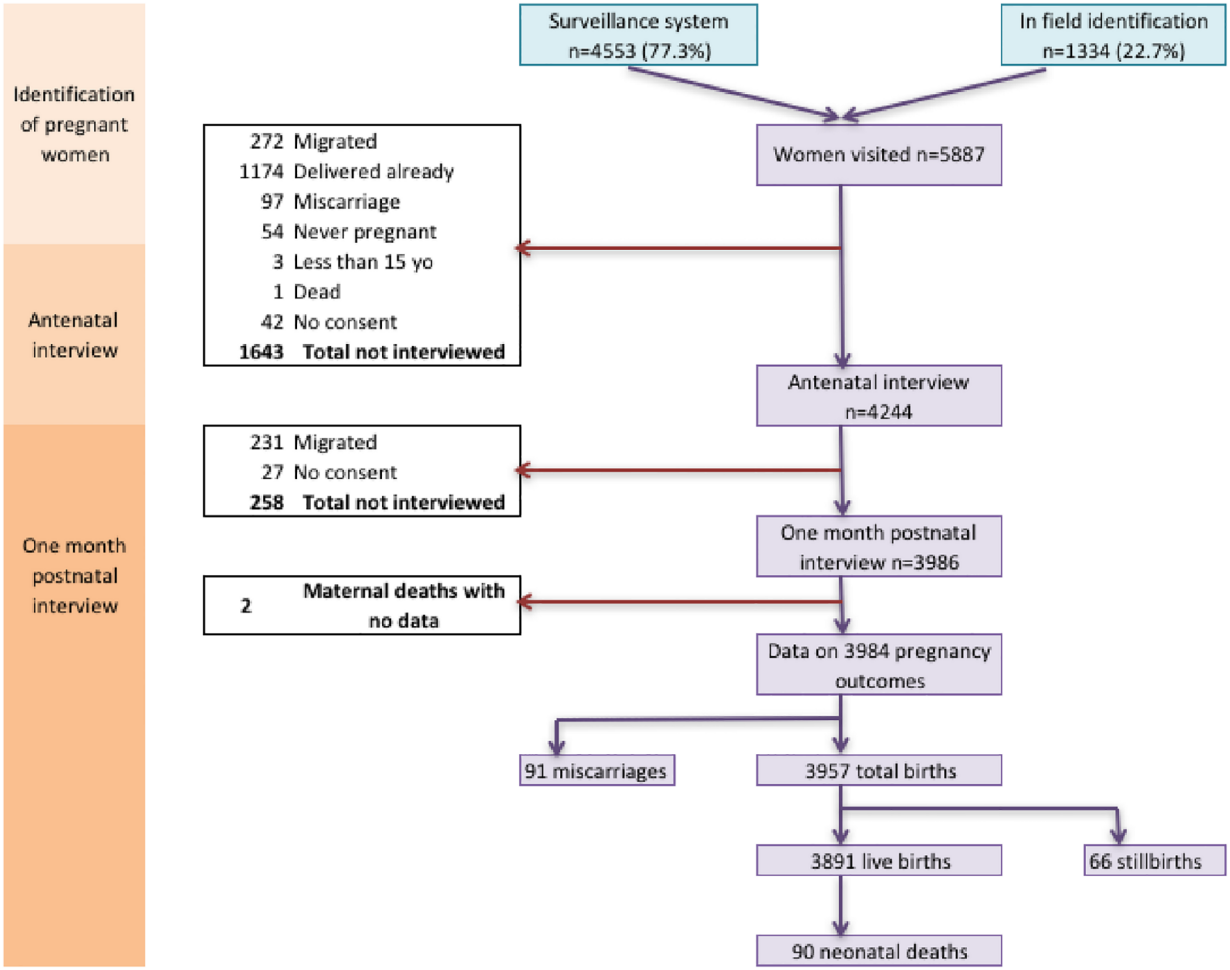
Case flow and outcomes for women recruited and followed up.

Of 5,887 pregnant women identified, 4,244 completed the antenatal interview. Women were between two and nine months pregnant (median six, mean 5.98) when interviewed. Fig 1 shows that 1174 (71·0%) of the women not recruited had already delivered when visited and were no longer eligible. Only 42 eligible women declined to participate, less than 1%. Such low refusal rates in our community-based recruitment suggest that these data are representative of the population of pregnant women in Mchinji District. The women recruited into the cohort have been described elsewhere [41] but, in brief, women were aged 15–49 (median 24), over 90% were married and 86% had no education or primary education only. Most women were Christians and were from the Chewa tribe. Women reported up to 15 previous pregnancies (median three) and 12 previous births (median two). Almost one in five women had experienced at least one child death and one in twenty had experienced at least one stillbirth, highlighting how common these adverse outcomes are in women’s lives.

3,986 women were followed up postnatally, as shown in Fig 1, a loss to follow-up of 258 women (6.08%), mostly due to migration. These women were not statistically significantly different with regards to their LMUP score, marital status, education, socio-economic status, parity (once adjusted for age) or birth interval, but were slightly younger (mean age 24·0 versus 25·1, p<0·01). The small loss to follow-up and lack of statistically significant differences suggests that the postnatal sample remains representative of pregnant women in Mchinji District. The median time since delivery (miscarriage, live or stillbirth) at postnatal interview was 40 days (IQR 27 to 79 days).

### Exposure and outcomes

The participants reported antenatal LMUP scores from zero to 12, reflecting the full range of intention.

As shown in Fig 1 there were 91 reported miscarriages (2·28% of pregnancies) leaving 3,893 pregnancies from which a total of 3,957 babies were born. Of these 66 were stillbirths, a rate of 16·7 per 1000 births [95%CI 12·9, 21·2], and 3,891 were live births. There were 90 early- or late-neonatal deaths, a neonatal mortality rate (NMR) of 23·1 per 1,000 live births [95%CI 18·6, 28·4], in keeping with other estimates at the time.

The average birthweight was 3·16kg (standard deviation 0·616g). 13·3% of babies were LBW (n = 526), in line with contemporary assessments. In total 18·0% of pregnancies (n = 717) resulted in an adverse outcome for the child.

Women’s postnatal SRQ score ranged from zero to eighteen (median one, IQR 0–3). 34.1% (n = 1357) reported no symptoms, 52.8% (n = 2101) reported low symptoms (1–5) and 13.0% (n = 518) reported high symptom levels (≥6).

**Fig 2:**
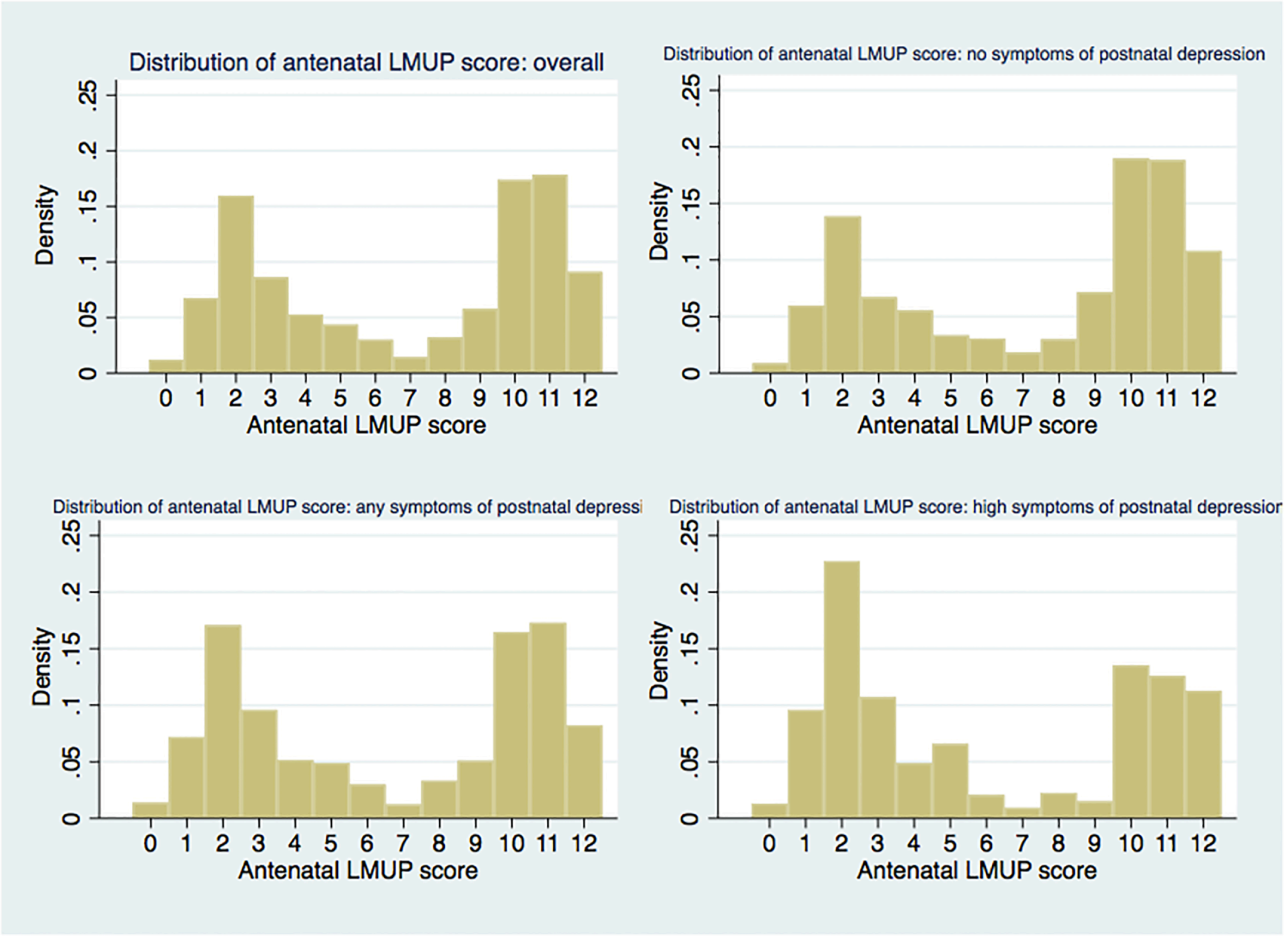
Distribution of antenatal LMUP score overall and by level of maternal postnatal depression symptoms.

**Fig 3:**
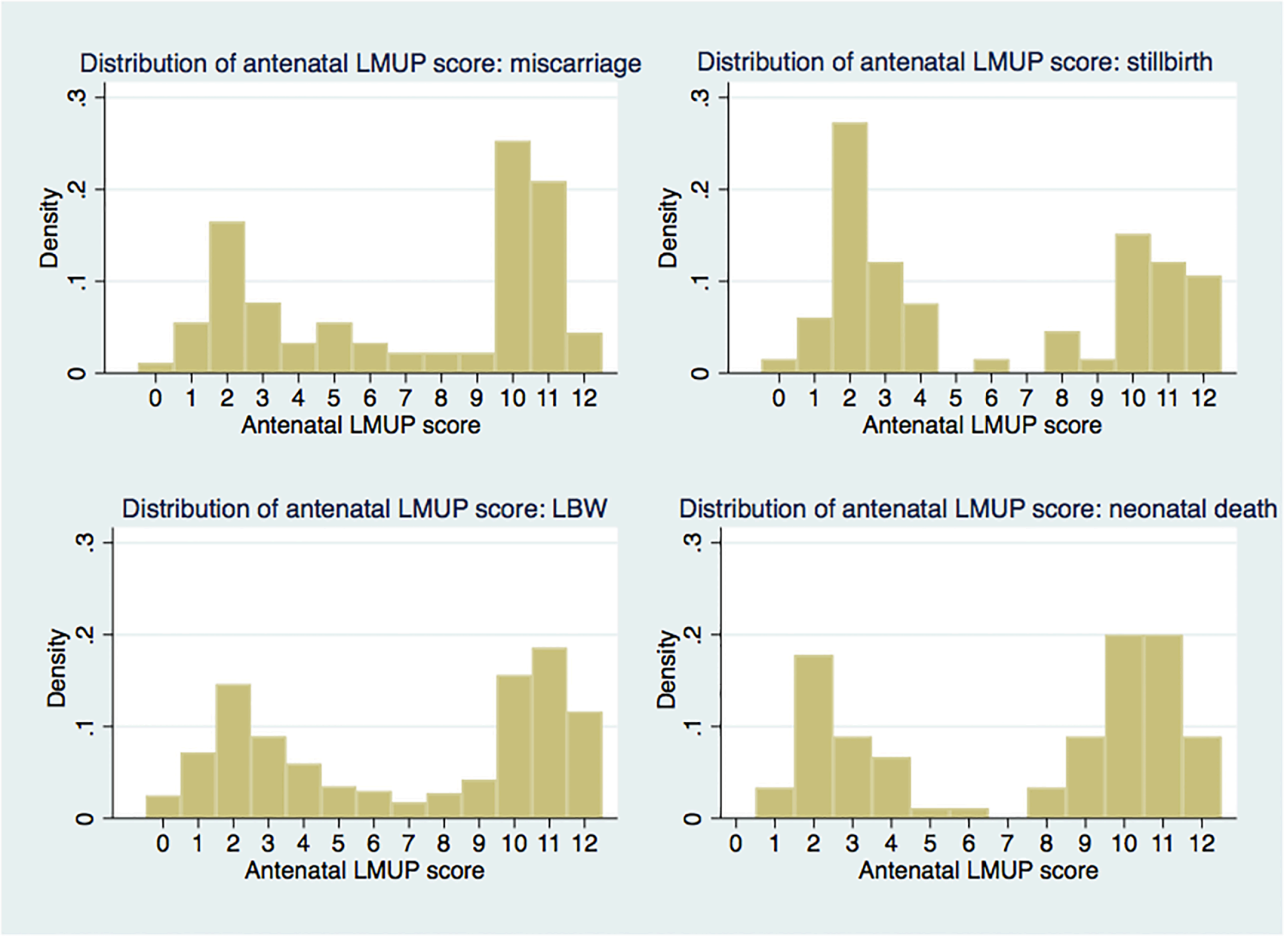
Distribution of antenatal LMUP score by miscarriage, stillbirth, low birthweight and neonatal death.

The overall distribution of the antenatal LMUP score and the distributions according to each adverse outcome are shown in Figs 2 and 3. These give a visual indication of the changing proportion of unplanned (antenatal LMUP score 0–3) and planned (antenatal LMUP score 10–12) pregnancies across the adverse maternal, perinatal and neonatal outcomes. The distribution of the antenatal LMUP score of the women not experiencing miscarriage, stillbirth, LBW and neonatal death is not presented as it is the same as the overall distribution given the small numbers that experienced each of these outcomes in the context of the size of the cohort.

### Pregnancy intention and postnatal depression

In univariate analysis greater pregnancy intention was significantly associated with a reduced risk of any symptoms of depression (SRQ≥1), RR 0.88 [95%CI 0.83, 0.92] for a planned compared to an unplanned pregnancy, as shown in Table 2.

**Table 2:** Univariate regression of pregnancy intention and any symptoms of depression.

	n (%) with any depression	RR	95% Conf.	Interval	p value
*Overall*	2619 (65.8)				
Pregnancy intention					<0.001
Unplanned	921 (71.3)	1	-	-	
Ambivalent	595 (64.7)	0.91	0.86	0.96	
Planned	1103 (62.5)	0.88	0.83	0.92	

All the determinants of pregnancy intention were included in a manual backwards stepwise model selection. Previous episodes of depression, sexual abuse and abuse in the last year remained significant, as shown in Table 3. Planned pregnancies remained associated with a significantly lower risk of any symptoms of depression compared to unplanned pregnancies (adjusted RR 0.90 [95%CI 0.86, 0.95]).

**Table 3:** Multivariable regression of pregnancy intention and any symptoms of depression.

	RR	95% Conf.	Interval	p value
Pregnancy intention				<0.001
Unplanned	1	-	-	
Ambivalent	0.92	0.87	0.97	
Planned	0.90	0.86	0.95	
Previous depression				<0.001
None	1	-	-	
1 or 2, <2 weeks	1.04	0.98	1.11	
One, > 2 weeks	1.15	1.08	1.21	
Both, > 2 weeks	1.09	0.91	1.30	
Abuse in the last year	1.15	1.07	1.23	<0.001
Sexual abuse	1.29	1.19	1.40	<0.001

Univariate analysis showed that increasing levels of pregnancy intention were significantly associated with a reduced risk of high levels of symptoms of depression (SRQ≥6), RR 0.62 [95%CI 0.52, 0.74] for a planned compared to an unplanned pregnancy, as shown in Table 4.

**Table 4:** Univariate regression of pregnancy intention and high symptoms of depression.

	n (%) with high depression	RR	95% Conf.	Interval	P value
*Overall*	518 (13.0)				
Pregnancy intention					<0.001
Unplanned	229 (17.7)	1	-	-	
Ambivalent	94 (10.2)	0.58	0.46	0.72	
Planned	195 (11.1)	0.62	0.52	0.74	

Once the determinants of pregnancy intention had been added to the model, the relationship between pregnancy intention and postnatal SRQ remained statistically significant (adjusted RR 0.76 [95%CI 0.63, 0.91] for a planned compared to an unplanned pregnancy). Intimate partner violence and previous episodes of depression all increased the risk of higher levels of symptoms of depression between 1.8 and 3.5 fold, as shown in Table 5. The risk of high symptom levels decreased with each additional year of maternal education and women having their first baby were at increased risk of having higher symptom levels.

**Table 5:** Multivariable regression of pregnancy intention and high symptoms of depression.

	RR	95% Conf.	Interval	p value
Pregnancy intention				<0.001
Unplanned	1	-	-	
Ambivalent	0.63	0.50	0.79	
Planned	0.76	0.63	0.91	
Maternal education (years)	0.96	0.93	0.99	0.003
Previous depression				<0.001
None	1	-	-	
1 or 2, <2 weeks	2.05	1.68	2.52	
One, > 2 weeks	2.49	2.04	3.03	
Both, > 2 weeks	3.56	2.36	5.38	
Abuse in the last year	1.83	1.48	2.27	<0.001
Sexual abuse	2.04	1.48	2.82	<0.001
First baby	1.37	1.14	1.64	0.001

### Pregnancy intention and composite adverse pregnancy outcome

There was no relationship between antenatal LMUP score and the composite adverse pregnancy outcome of miscarriage, stillbirth, low birthweight and neonatal death (RR 1·00 [95%CI 0·99, 1·02]) on univariate analysis.

### Pregnancy intention and stillbirth

There was some evidence of a broadly linear relationship between antenatal LMUP score and stillbirth on univariate regression so we used the full antenatal LMUP score in the analysis. The risk of stillbirth was reduced for every one point increase in the degree of pregnancy intention, RR 0·94 [95%CI 0·89, 1·00] p = 0.055 on univariate analysis. An exploratory analysis was pursued.

After manual backwards step-wise model selection the only variable retained was primiparity, as shown in Table 6, and the adjusted risk ratio between antenatal LMUP score and stillbirth was 0·93 [95%CI 0·87, 1·00] p = 0.04) becoming statistically significant.

**Table 6:** Multivariable regression of pregnancy intention and stillbirth.

Stillbirth				
	RR	95% Conf. Interval		P value
Antenatal LMUP score (per unit)	0.93	0.87	1.00	0.04
First baby	1.80	1.03	3.15	0.04

This suggests that for every one-point increase in antenatal LMUP score (greater intention) there is an adjusted risk of stillbirth of 0·93. Across the range of the antenatal LMUP score, a pregnancy with an antenatal LMUP score of 12 would have 0·42 times the risk [95%CI 0·19, 1.00] of having a stillbirth than a pregnancy with an antenatal LMUP score of zero.

### Pregnancy intention and other individual adverse pregnancy outcomes

There was no relationship between antenatal LMUP score and miscarriage (RR 1·01 [95%CI 0.96, 1.06]), LBW (RR 1·00 [95%CI 0·98, 1·02]), or neonatal death (RR 1·03 [95%CI 0·98, 1·08]) on univariate analysis.

Sensitivity analyses of the relationship between antenatal LMUP score and postnatal SRQ score using the non-imputed data confirmed our findings. For each of the relationships between antenatal LMUP score and the composite pregnancy outcome, miscarriage, low birth weight and neonatal death we checked for negative confounding, which was not present.

## Discussion

### Main findings

Our analyses have found that planned pregnancies are associated with a reduced risk of any (adjusted RR 0.90 [95%CI 0.86,0.95]) or high symptoms of depression (adjusted RR 0.76 [95%CI 0.63, 0.91]) compared to unplanned pregnancies in rural Malawi. Many studies have identified unplanned pregnancy as a risk factor for postnatal depression in high income countries (see, for example, [42–44]) however there are far fewer data from low income countries and the findings have been mixed [45].

Having grouped the antenatal LMUP score into the recognised categories of unplanned, ambivalent and planned, we can see from our analyses that there is a suggestion of a dose-response relationship between the level of pregnancy intention and the risk of any symptoms of postnatal depression in both the uni- and multi-variate models (Tables 2 and 3). Though the difference in risk between ambivalent and planned pregnancies is small and not statistically significant, the direction of the relationship provides further evidence of the validity of the measures of both pregnancy intention and depression. The picture is less clear when we compare none/any symptoms with high symptom levels. Here there is a suggestion that women with ambivalent pregnancies have a lower risk of high symptom levels than women with planned pregnancies. This difference is not statistically significant and it should be bourne in mind that the number of women experiencing high symptom levels was low (13%, n = 518), and that within this the proportion reporting ambivalent pregnancies was also small (18%, n = 94), meaning that these findings are less precise.

Our robust methodology, collecting the data prospectively, using validated measures of both pregnancy intention and postnatal depression and carefully selecting confounders based on a conceptual epidemiology hierarchy [41] lends considerable rigour to these findings. The fact that we found a suggestion of a dose-response relationship between level of pregnancy intention and risk of postnatal depression, and that the effect sizes were greater for high symptom levels than any symptoms also provides further evidence of the validity of the assessment of pregnancy intention and postnatal depression. We would not have been able to look for such a relationship had we used the DHS question, as this only looks as the issue of the timing of the pregnancy, and not an overall assessment of the degree of pregnancy intention. Furthermore, the DHS question tends to overestimate the level of pregnancy intention due to recall bias of ex-post rationalisation [16, 17], which may have prevented us from observing the relationships between the lower levels of intention (mistimed and unwanted in DHS-terms) due to smaller numbers.

More planned pregnancies were associated with a significantly reduced risk of stillbirth on univariate analysis which was slightly enhanced in the multivariable model. These data suggest that a less planned pregnancy is potentially an important contributory risk factor to stillbirth in low-income countries. Only one study has previously investigated this, where it was found that unplanned pregnancies had about double the risk of ‘pregnancy loss’ (miscarriage, induced abortion or stillbirth) [46]. This potential relationship should be investigated in larger prospective cohorts.

Until recently, stillbirths have been a neglected topic in global health. Our research suggests that addressing unplanned pregnancy may have a role to play in tackling stillbirths in low resource settings. This could be via primary prevention of unplanned pregnancy through improving access, uptake, continuation and quality in family planning services, by identifying women planning a pregnancy and providing health checks, eg for syphilis and other sexually transmitted infections associated with fetal loss, and by education of healthworkers on the importance of the preventing unplanned pregnancy for improving maternal and perinatal health. There is also a role for secondary prevention by identifying women with a current unplanned pregnancy and supporting them to access appropriate antenatal and delivery services to reduce stillbirths.

The lack of relationship between pregnancy intention and miscarriage, LBW and neonatal mortality was not what was expected from the literature, particularly for LBW. This could be because most published literature is from high-income countries and there may be genuine differences in the relationships between pregnancy intention and outcomes in different settings, or the determinants of LBW may vary. For example in low-income countries there is often greater food insecurity, lower maternal height and poorer gestational weight gain, which may overwhelm any potential impact of pregnancy intention on birthweight. In this study setting, 54% of children under the age of five are stunted [25] and over half of women in the study said they ‘never’ or ‘rarely’ had enough to eat; in this context there are clearly much more important determinants of birthweight than pregnancy intention. Alternatively it could be due to the bias introduced by the methodologies used in other studies. Replicating our study methodology in a high-income country would help to determine whether the relationship previously seen in high-income countries is real or an artefact of the study design.

### Strengths and limitations

This research has overcome many of the methodological limitations of previous studies in this area. This is the first study to investigate prospectively the relationship between pregnancy intention, assessed by a robust measure with established psychometric properties (the LMUP), and subsequent maternal and neonatal outcomes. Our research also contributes to filling gaps in the literature by examining understudied outcomes, such as miscarriage and stillbirth, in a low-income country setting.

The main limitation, which this research shares with other studies, is the lack of information about early miscarriages and induced abortions. This will result in an underestimate of unintended pregnancy and its effect on adverse pregnancy outcome, particularly as most abortions in Malawi and other low resource settings will be unsafe. The lack of a reliable way of assessing gestation in rural Malawi also meant that pre-term birth could not be studied.

While this study was prospective, we have not conducted a causal or mediation analysis and therefore cannot comment on *how* pregnancy intention is associated with adverse outcomes. Uptake of care and other antenatal, delivery and postnatal factors may be mediators, in which case some of the effect of unintended pregnancy could be mitigated through preventative services. Further studies and analyses are required to investigate this.

## Conclusions and implications

Our research has shown that a robust, simple and sensitive scale (LMUP) can be used to identify women with less planned pregnancies who are at increased risk of postnatal depression and, possibly, stillbirth. This implies that implementing the LMUP into routine maternity care would improve identification of women at increased risk of adverse outcomes. Having identified these women, the healthcare worker could provide additional counselling and support to enable them to access adequate care and preventative practices. The antenatal LMUP score could be used to flag women who are at risk of postnatal depression and who should be assessed after birth. While provision of mental health services is often limited in low income countries, following basic training healthcare workers have been shown to be competent to diagnose common mental health problems and either initiate treatment or refer to specialist services as appropriate [47]. Until universal screening for postnatal depression is introduced, this targeted approach should not overburden healthcare workers but would facilitate the detection of more women at risk of postnatal depression.

Our findings provide evidence of the relevance of the issues of pregnancy planning, postnatal depression and stillbirth to low resource settings and can be used to inform context specific reproductive, maternal and child health policy.

## Supporting information

S1 Table Factors considered for inclusion in the multiple imputation model for missing low birthweight data. (DOCX)
