# Supplementary material for: Reassessing pregnancy intention and its relation to maternal, perinatal and neonatal outcomes in a low-income setting: A cohort study
Source: PLoS One. 2018 Oct 18;13(10):e0205487. doi: 10.1371/journal.pone.0205487 (PMC6193645; doi:10.1371/journal.pone.0205487)
Supplement: S1 Table — (DOCX) [file pone.0205487.s001.docx]

| **Socio-demographic / economic** | **Parental anthropometry** | **Obstetric history** | **Antenatal factors** | **Delivery factors** | **Infant factors** | **Infant postnatal factors** |
| --- | --- | --- | --- | --- | --- | --- |
| Socio-economic status^1^ | Maternal height^1^ | Parity^1^ | ANC uptake^1^^ | Location^2^ | Sex^1^ | Weight at postnatal visit^6^ |
| Maternal age^1^ | Pre-pregnancy weight^3^ | Birth interval^1^ | Morbidity^1^+ | Person^2^ | Maternal estimate of size^1^ | Length at postnatal visit^6^ |
| Maternal education^1^ | Paternal height^3^ | History of LBW baby^3^ | Malaria^3^ |  | Multiple birth^1^ | Age at postnatal visit^6^ |
| Paternal education^1^ | Paternal weight^3^ | Number of live children^2^ | Gestational weight gain^3^ |  | Maternal estimate of gestation_1_ | Alive at postnatal visit^6^ |
| Marital status^1^ | Maternal birth weight^3^ |  | Caloric intake^3^ |  |  |  |
| Intimate partner violence^1^ | Maternal MUAC^4^ |  | Iron/folic acid use^4^ |  |  |  |
| Safe water^1^* |  |  | IPTp^4^ |  |  |  |
| Rural area^1^** |  |  | Smoking^5^ |  |  |  |
| Ethnicity^5^ |  |  | Alcohol^5^ |  |  |  |
| Previous depression^2^ |  |  | LMUP^2^ |  |  |  |
|  |  |  |  |  |  |  |
|  |  |  |  |  |  |  |
| **^1^ Measured and included** | **^2^ Relevant to main analysis** | **^3^ Data not collected** | **^4^ Proxy for data not collected** | **^5^ Not applicable** | **^6^Postnatal factors used in imputation model** | |
|  |  |  |  |  |  |  |
| * included in measure of SES | |  | ^ measured by months at booking and total attendances | | |  |
| ** cluster variable included, also captured in measure of SES | | | + reported antenatal problems (any, including malaria) and antenatal SRQ score | | | |
